# Welcome to Discover Social Science and Health

**DOI:** 10.1007/s44155-021-00002-w

**Published:** 2021-04-06

**Authors:** Michael J. Shanahan

**Affiliations:** University of Zürich, Zürich, Switzerland

On behalf of the entire editorial team, I am pleased to inaugurate *Discover Social Science and Health* (*DSSH*), which will showcase research that bridges the social and biomedical sciences.

The launch of this new journal is especially appropriate in the midst of the COVID-19 pandemic, which has vividly revealed, to lay-person and scientist alike, how profoundly the combination of social and biological factors shapes human health. The story of COVID-19 is complex, but already we see (among other things) how infections, vaccinations, hospitalizations, and deaths reflect geopolitical forces, national and regional policies, spatial diffusion mechanisms, and myriad forms of racial and ethnic disparities. Yet to scientists working at the intersection of the social and biomedical sciences, it is abundantly clear that, like COVID-19, other diseases and disease processes, symptoms, treatments, and outcomes also reflect such mechanisms.

In addition to its obvious topicality, the launch of *DSSH* offers several other grounds for enthusiasm. First, *DSSH* is inclusive of all branches of the social sciences and all types of data that describe human health, which is broadly conceived to include indicators of general health, diagnoses, symptoms, health care, treatment adherence, and sequelae. This inclusion reflects the conviction that diverse theoretical and empirical approaches collectively inform larger scientific questions related to inequalities in health and to the social organization of health. *DSSH* is thus a new forum that will promote cross-talk among scientists studying similar topics from differing perspectives.

Second, *DSSH* will publish papers that are at the intersection of social and biomedical sciences. This bridging is made possible by many exciting developments, including the collection of sophisticated forms of health data in social surveys, big data efforts that join medical records to other forms of data, and a growing list of training initiatives that introduce social scientists to medical, biological, and other forms of health data. In turn, these developments warrant new spaces for increasingly sophisticated, interdisciplinary research that joins social and health data.

Third, *DSSH* offers diverse formats for submissions, and we hope that these new opportunities enliven the field. These formats include traditional papers but also Perspectives, Reviews, Brief Communications, and Data Notes—new formats that will create a one-of-a-kind forum to share findings, express viewpoints, and suggest ways forward in both theory and data analysis. *DSSH* will publish solid replications, pre-registered studies, and well-executed studies—whatever the findings.

Finally, Springer Nature has innovated its publishing model with the new Discover series. The submission and review process will be expedited, while still ensuring robust and independent peer-review for all publications, and DSSH will be open-access with competitive fees. While open-access is not in itself innovative, many scientists believe that the trend toward open-access should continue.

For these and other reasons, the editorial team is excited by this new journal, and we will strive to realize these ambitions fully. Above all, we hope that this new journal will promote understanding of how the social and biomedical join to affect human health and, in turn, of how these insights can translate into better health for everyone.

